# Discordance in cathepsin B and cystatin C expressions in bronchoalveolar fluids between murine bleomycin-induced fibrosis and human idiopathic fibrosis

**DOI:** 10.1186/s12931-016-0432-6

**Published:** 2016-09-22

**Authors:** Mariana Kasabova, Bérengère Villeret, Aurélie Gombault, Fabien Lecaille, Thomas Reinheckel, Sylvain Marchand-Adam, Isabelle Couillin, Gilles Lalmanach

**Affiliations:** 1 INSERM, UMR 1100/Centre d’Etude des Pathologies Respiratoires (CEPR), Université François Rabelais, Faculté de Médecine, 10 Boulevard Tonnellé, F-37032 Tours, cedex France; 2 CNRS, UMR-INEM 7355, Immunologie et Neurogénétique Experimentales et Moléculaires, Université d’Orléans, Orléans, France; 3 Institute of Molecular Medicine and Cell Research, Medical Faculty, Albert-Ludwigs-University Freiburg, Freiburg, Germany; 4 Centre Hospitalier Régional Universitaire Tours, Service de Pneumologie, Tours, France; 5 Present address: Laboratoire de Biologie des Tumeurs et du Développement (LBTD), Université de Liège, Liège, Belgium

**Keywords:** Bronchoalveolar lavage fluid, Bleomycin, Cathepsins, Cystatins, Fibrosis, Lung, Proteases

## Abstract

The activity of cysteine cathepsin B increased markedly in lung homogenates and in bronchoalveolar lavage fluids (BALF) of the mouse model of bleomycin-induced lung fibrosis after 14 days of challenge. In contrast the level of the cysteine cathepsin inhibitor cystatin C was unaffected in BALF of wild-type and cathepsin B-deficient mice. Therefore, murine cystatin C is not a reliable marker of fibrosis during bleomycin-induced lung fibrosis. Current data are in sharp contrast to previous analysis carried on human BALF from patients with idiopathic pulmonary fibrosis, for which the level of cathepsin B remained unchanged while cystatin C was significantly increased.

## Findings

### Cathepsin B, cystatin C and idiopathic pulmonary fibrosis: A quick focus

Idiopathic pulmonary fibrosis (IPF) is a chronic irreversible lung disease of unknown etiology characterized by an important deposition of extracellular matrix (ECM) components in the interstitial space and alveoli [1]. Most of in vivo studies relied on the murine model of bleomycin (BLM)-induced lung fibrosis [2]. Besides irradiation, fluorescein isothiocyanate (FITC) or silica model, the bleomycin model is the best characterized model with common characteristics of human IPF and is considered to be clinically relevant. Moreover, the experimental time frame is short and reproducible with the development of fibrosis occurring by day 14 (D14) as seen both biochemically and histologically [2]. However, fibrotic mechanisms for IPF and bleomycin-induced lung damage may be different, since experimental lung fibrosis is induced by a single dose of bleomycin applied via the intratracheal route and the role of inflammation is crucial, while human IPF is the result of repeated and diverse aggressions throughout the patient’s life and inflammatory episodes are of secondary importance. Moreover in the murine model, fibrosis is reversible as opposed to IPF that is a progressive and irreversible process leading to respiratory failure.

Cysteine cathepsins (11 members in humans) are proteases participating actively in ECM remodeling and in fibrotic disorders [3]. Cathepsin B (CatB) may contribute to lung myofibrogenesis by triggering the TGF-β1-driven Smad 2–3 pathway [4]. Conversely, pharmacological inhibition and genetic silencing of CatB diminished α-SMA expression, delayed fibroblast differentiation and led to an accumulation of intracellular pro-TGF-β1. In addition CatB drives activation of hepatic stellate cells, and participates in liver fibrogenesis. Recently, a conclusive trial (phase I) for the treatment of hepatic fibrosis (VBY-376, a CatB inhibitor from Virobay, Menlo Park, Ca, USA) supported the notion that the use of CatB inhibitors could be appropriate for therapy of lung fibrosis.

The proteolytic activity of cysteine cathepsins is regulated by their natural endogenous inhibitors belonging to the cystatin family [5, 6]. Among this family, cystatin C is the most potent inhibitor of cysteine cathepsins. Cystatin C is synthesized and secreted by an extensive variety of human cells with a widespread distribution in body fluids and tissues [7]. A significant increase of immunoreactive cystatin C was found in human bronchoalveolar lavage fluids (BALF) from IPF patients, raising the question of its use as a potential marker of IPF [8]. On the other hand, mouse and human cystatin C share 71 % of identity on amino acid level implying highly similar structural and functional properties of the homologues. Mouse cystatin C is expressed by all tissues with a relative content very similar to that of human tissues and has a widespread distribution in body fluids [9]. In terms of regulation cystatin C is rather seen as a housekeeping gene, because previous work could not establish its transcriptional regulation in response to various stimuli (e.g. bacterial and viral infections, stress, cytokines, growth factors) [10].

### Increase of CatB activity in both lung homogenates and bronchoalveolar lavage fluids

**Fig. 1:**
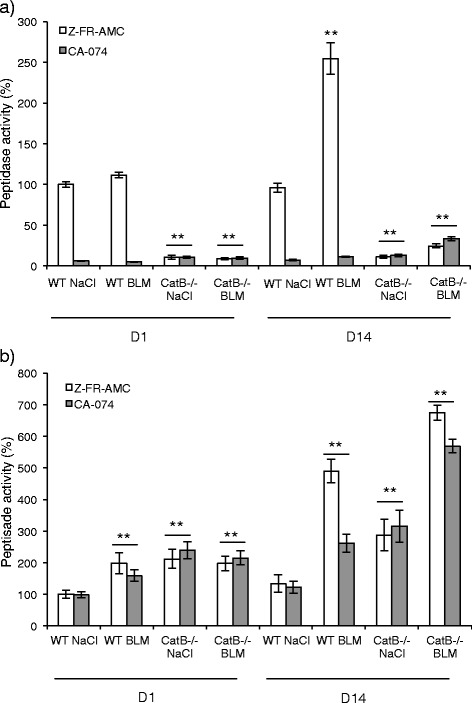
Peptidase activity of cysteine cathepsins after bleomycin administration to wild type and cathepsin B-deficient mice. Mice (C57BL/6 strain) were challenged with BLM (7.5 mg/kg for day 1; 3 mg/kg for day 14) or saline solution by intranasal instillation and sacrificed after 1 and 14 days [19]. Lung homogenates and BALF were prepared as described earlier by Gasse et al. ([19]) and Kasabova et al. ([8]). **a** Lung homogenates and (**b**) BALF were incubated in the activity buffer (0.1 M sodium acetate pH 5.5, 2 mM DTT, 2 mM EDTA, 0.01 % Brij35) for 5 min. at 30 °C. The overall cathepsin activity was measured toward Z-FR-AMC (50 μM, λ_exc_ = 350 nm and λ_em_ = 460 nm) at day 1 (D1) and day 14 (D14) (Gemini spectrofluorimeter, Molecular Devices). *White* bars: endopeptidase cathepsin activity. *Grey* bars: control experiments. Samples were preincubated in the presence of CA-074 (10 μM) for 30 min prior to measuring the peptidase activity. Data are expressed as mean values ± SEM. **p < 0.05 (Kruskall and Wallis non-parametric test) (WT NaCl, *n* = 5; WT BLM, *n* = 6; CatB^-/-^ NaCl, *n* = 5; CatB^-/-^ BLM, *n* = 8)

BALF and lung homogenates from wild type (C57BL/6 strain) and CatB-deficient (CatB^-/-^) mice [11] were collected at D1 or D14 post-BLM treatment. To our knowledge consequences of the genetic deletion of CatB in bleomycin-induced fibrosis have not been reported or studied elsewhere. In this study, CatB^-/-^ mice served primarily as control for assessment of the CatB-dependent peptidase activity. Samples were handled as described previously [8]. At D1, the overall cathepsin activity in lung homogenates remained unchanged for BLM- and saline-treated WT mice (Fig. 1a). Pre-incubation with CA-074, a selective CatB inhibitor [12], showed that CatB is the prevailing active cathepsin. Consistently, the peptidase activity decreased dramatically (~85 %) in lungs from saline- and BLM-treated CatB^-/-^ mice. At D14, BLM instillation resulted in a 3-fold increase of the cathepsin activity in WT mice. Addition of CA-074 indicated that this activity is mostly CatB-dependent. Again cathepsin activity in BLM-treated CatB^-/-^ mice was markedly decreased (~80 %), confirming that BLM administration to WT mice induces an overexpression of CatB at D14 (Fig. 1a). Results correlate with a recent article uncovering an increase of lung cathepsins after BLM administration [13]. In this elegant study, the use of an optical probe revealed a specific and maximal labeling (D14) of cathepsins at sites of fibrotic lesions correlated with the extent of disease burden [13].

Cysteine cathepsin activity was next considered in BALF. At D1 post-BLM treatment, a ~2-fold increase of the activity was observed in BLM-treated WT mice compared to saline control. Pre-incubation with CA-074 demonstrated that active CatB, in contrast to the measurements in lung homogenates, is not the major cathepsin found in BALF at D1. The equivalent overall cathepsin activity in BALF from CatB^-/-^ saline control and BLM-treated CatB^-/-^ mice corroborated this statement (Fig. 1b). A 4-fold increase of cathepsin activity was assessed for WT mice at D14 post-BLM treatment and ~50 % of the peptidase activity related to CatB contrary to that observed at D1. Moreover, unlike lung homogenates, the cathepsin activity increased ~6-fold in CatB^-/-^ mice after BLM challenge (Fig. 1b), supporting functional redundancy and/or the establishment of compensatory mechanisms between cysteine cathepsins [14, 15]. Present results differ from those observed in human BALF where no significant difference in cathepsin activity, including CatB, was observed between non-fibrotic and IPF patients [8]. A key point is that a similar amount of macrophages, which are the primary source of cysteine cathepsins in BALF, was found for both groups of patients [8]. Conversely in the murine BLM-induced lung fibrosis activated macrophages (with a M2/M2-like phenotype) are predominant in the immune infiltrate and BALF at D14, in association with the presence of markers of the Th2 profibrotic response [13]. Nevertheless recruited macrophages have intermediate proinflammatory and profibrotic phenotypes [16] and in overall the changes in the levels of key cytokines and chemokines upon bleomycin-induced fibrosis were consistent with those observed in human IPF [17].

### The concentration of cystatin C is not affected by BLM challenge

**Fig. 2:**
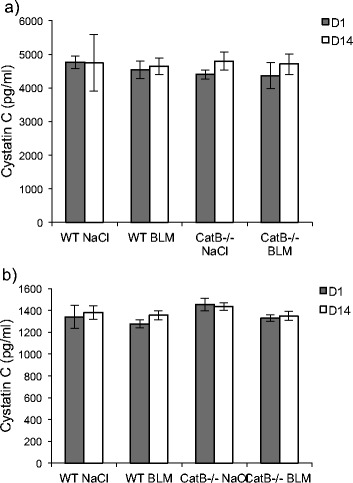
Cystatin C immunoassay. Dosage of immunoreactive cystatin C was performed after BLM (or saline solution) administration to wild-type and CatB-deficient mice (see legend to Fig. 1). The concentration of cystatin C was determined using the mouse/rat ELISA Quantikine kit according to the manufacturer’s instructions (R&D Systems Inc, Minneapolis, MN, USA). **a** Lung homogenates and (**b**) BALF. Data are expressed as mean values ± SEM (WT NaCl, *n* = 5; WT BLM, *n* = 6; CatB^-/-^ NaCl, *n* = 5; CatB^-/-^ BLM, *n* = 8)

We reported earlier a significant increase of immunoreactive cystatin C, in human BALF from IPF patients raising the question of its potential use as a new biomarker [8]. The increase of cystatin C level was significant for each of three IPF severity grades (stages I, II, II). Cystatin C has long been validated besides creatinine as a serum biomarker of glomerular filtration rate, but we did not measure a statistically confident variation in alveolar concentration of cystatin C between patients with a low (<60 ml/min) or a high (>60 ml/min) glomerular clearance (*p* > 0.1) and no co-morbidity in conjunction with cystatin C level was observed [8]. Here, cystatin C concentration remained unchanged both in lung extracts (Fig. 2a) and BALF (Fig. 2b) for BLM-treated and control mice at D1 and D14. The same level of BALF cystatin C was also measured for saline- and BLM-treated CatB^-/-^ mice. Conversely to that observed in human BALF from IPF patients, cystatin C level is unaffected at D14 following BLM challenge, supporting clearly that murine cystatin C cannot be embraced as a marker in BLM-induced lung fibrosis.

Of interest bronchopulmonary dysplasia (BPD) is characterized by impaired alveolar development and widespread bronchial disease, and consecutive fibrotic changes are observed. Results in accordance with the present study were reported using a non-human primate model of BPD: mRNA and protein levels of CatB were significantly increased in the lung tissue of baboons with BPD. In contrast, both mRNA and protein levels of cystatin C remained unchanged in lung tissue lysates and BALF [18]. Although the rodent model of BLM-induced fibrosis is of mandatory concern to decipher proteolytic mechanisms of fibrogenesis, the present data confirm and point out that transposition of the results to human IPF should be done with caution.

## Abbreviations

AMC: 7-Amino-4-methylcoumarin
BALF: Bronchoalveolar lavage fluid, BLM, Bleomycin
CA-074: N-(L-3-trans-propylcarbamoyloxirane-2-carbonyl)-L-isoleucyl-L-proline
Cat: Cathepsin
ECM: Extracellular matrix
IPF: Idiopathic pulmonary fibrosis
WT: Wild type
Z: Benzyloxycarbonyl
